# The Association between Splenocyte Apoptosis and Alterations of Bax, Bcl-2 and Caspase-3 mRNA Expression, and Oxidative Stress Induced by Dietary Nickel Chloride in Broilers

**DOI:** 10.3390/ijerph10127310

**Published:** 2013-12-17

**Authors:** Jianying Huang, Hengmin Cui, Xi Peng, Jing Fang, Zhicai Zuo, Junliang Deng, Bangyuan Wu

**Affiliations:** Key Laboratory of Animal Diseases and Environmental Hazards of Sichuan Province, College of Veterinary Medicine, Sichuan Agricultural University, Ya’an 625014, China; E-Mails: hjy19860316@163.com (J.H.); pengxi197313@163.com (X.P.); fangjing4109@163.com (J.F.); zzcjl@126.com (Z.Z.); dengjl213@126.com (J.D.); wubangyuan2008@163.com (B.W.)

**Keywords:** NiCl_2_, apoptosis, apoptosis protein, mRNA expression, oxidative stress, qRT-PCR, FCM, broiler

## Abstract

Two hundred and forty avian broilers were equally divided into four groups, and raised with a corn-soybean basal diet or the same diet supplemented with 300, 600, 900 mg/kg NiCl_2_ for 42 days. Numbers or percentages of apoptotic splenocytes by flow cytometry (FCM) and TUNEL were higher (*p* < 0.05 or *p* < 0.01) in the 300, 600 and 900 mg/kg groups than those in the control group. Results measured by qRT-PCR and ELISA showed that mRNA expression and contents were significantly higher (*p* < 0.05 or *p* < 0.01) in Bax and Caspase-3, and were significantly lower (*p* < 0.05 or *p* < 0.01) in Bcl-2 of the 300, 600 and 900 mg/kg groups. Also, the SOD, CAT and GSH-Px activities, and the ability to inhibit hydroxyl radical, and GSH contents were significantly decreased (*p* < 0.05 or *p* < 0.01), and MDA contents were increased (*p* < 0.05 or *p* < 0.01) in all groups. In conclusion, dietary NiCl_2_ in excess of 300 mg/kg caused apoptosis, altered Bax, Bcl-2 and Caspase-3 mRNA expression levels and contents, and induced oxidative stress in the spleen. Also, splenocyte apoptosis was closely related to the alternations of Bax, Bcl-2 and Caspase-3 mRNA expression, and oxidative damage. The splenic immunity and blood filtration functions were impaired in broilers.

## 1. Introduction

Nickel (Ni) is a prevalent metal in the ecosystem of both geographical distribution and human activities. It exists as an essential element of more than one hundred compounds, and it was widely used in industry and commerce [1,2,3]. Naturally, the most common oxidative state of Ni is the +2 valence, and Ni salts such as nickel chloride (NiCl_2_), sulphate, nitrate, carbonate, hydroxide, acetate and oxide are valuable in commerce [4,5,6]. Water-soluble Ni salts such as NiCl_2_, nitrate, sulphate are easily absorbed [2,7,8]. Ni gets into human and animals mainly through inhalation, drinking water and food, and among these pathways, food are the most important [2]. 

Ni is considered as an essential metal nutrient for many species [9,10,11]. It has been reported that Ni is vital for some hormones (like prolactin, adrenaline, noradrenaline and aldosterone) and proper function of the immune system, and its deficiency will inhibit growth, reduce reproductive rate and alter glucose and lipid metabolism, and intracellular Ni can change membrane properties and influence ox/red systems [12,13,14,15]. Depigmentation of the skin, thickened legs, swollen hocks, growth retardation and anemia has been reported in chicks fed on a Ni deficient diet [15]. However, Ni and its compounds could also be toxic to human beings and animals [1,2,3]. Previous studies have proved that long-term exposure to Ni is deleterious to the upper respiratory tract, skin, kidney, immune system [16,17,18,19], embryos, and the breeding system [8,20,21]. Even short-term exposure to Ni can significantly influence the cardiovascular system [18,19]. Ni in the body can also disturb the metabolic balance of other elemental metals, which suppresses the toxicity and carcinogenicity of nickel. Nickel toxicity mainly results from the ability to replace other metal ions in enzymes and proteins or to bind to cellular compounds containing O-, S-, and N-atoms [4,22,23]. 

It had been reported that Ni can enhance lipid peroxidation (LPO) and cause cellular damage and reduced glutathione (GSH) contents, and catalase (CAT) and glutathione peroxidase (GSH-Px) activities in the liver and kidney of rats [24,25]. Ni-ion accumulation may be responsible for the generation of reactive oxygen species (ROS) and the enhancement of LPO, and NiCl_2_ is related to DNA oxidation and DNA strand breaks in rat’s liver [23]. NiCl_2_-induced human lymphocyte toxicity may be mediated by oxygen radical intermediates [26]. Our earlier study has proved that NiCl_2_ can cause oxidative damage in the intestinal tract of broiler [27]. Data also show that oxidative stress caused by Ni is important in the mechanism of Ni toxicity [24,25,28,29,30]. 

Previous studies show that Ni can cause DNA damage, inhibit DNA repair in mammalian cells [31,32,33]. Some articles describe that Ni can induce cell apoptosis, and the mechanisms have been well documented. Ni (II) can directly generate ROS, activate Caspase-3 expression, increase Caspase-3-like protease activity, and then cause cell death in mice [34,35]. It has been also reported that nickel ferrite nanoparticles-induced oxidative stress mediates apoptosis in cultured A549 cells [36].

Spleen is a peripheral immune organ in the body. Ni is considered to be toxic to the immune system. Ni (II) exposure has multiple effects on the immune system, including thymic involution, decreasing T cell number and natural killer cell activity in the spleen of mice [35]. A previous article has reported that Ni (II) can reduce cell viability and proliferation of Jurkat T cells, which are similar to human T lymphocytes [34]. Animal studies show that NiCl_2_ can accumulate in the spleen of mice [29], and promote immunosuppression [37]. Ni could accumulate in the spleen and cause changes both in number and size of the giant cells [38,39]. NiCl_2_ and NiS can also influence cellular proliferation in the spleen [40,41]. Studies on CBA/J and C57BL/6J mice show that NiCl_2_ significantly reduced the NK cell activity in the spleen of mice [42,43], but the mechanisms of the effects of Ni compounds on splenocytes are still unknown [44]. 

At present, studies on NiCl_2_ in chickens mainly focus on bone of male broiler, egg productivity, feather and fatting programs, and biochemical parameters in liver and blood [45,46,47], and very limited research focuses on the effects of NiCl_2_ on the splenic apoptosis and oxidative stress in animals and human beings. 

Taking previous research on laying hens [48], the concentration in the environment [49], and our preliminary experiment into consideration, we choose to add dosages of 300, 600 and 900 mg/kg NiCl_2_ to the basal diet of broilers and then we attempted to systematically investigate how Ni induced apoptosis and oxidative stress in the spleen, and the association between these effects in broiler. Thus, the activities of superoxide dismutase (SOD), CAT and GSH-Px, and the ability to inhibit hydroxyl radical, and GSH and malondialdehyde (MDA) contents were used to measure the oxidative stress in the spleen, and mRNA expression and contents of Bax, Bcl-2 and Caspase-3, and apoptotic cells detected by ELISA, qRT-PCR, terminal dexynucleotidyl transferase (TdT)-mediated dUTP nick end labeling (TUNEL) and FCM were used to investigate the apoptotic condition in the spleen caused by NiCl_2_.

## 2. Materials and Methods

### 2.1. Chickens and Diets

These were described previously [27]. Two hundred and forty one-day-old healthy avian broilers were randomly divided into four groups by body weight with 60 broilers in each group. Broilers were housed in cages with electrical heater and were provided with diets and water *ad libitum* for 42 days. A corn-soybean basal diet formulated by the National Research Council (NRC) [50] was the control diet. NiCl_2_ was mixed into the corn-soybean basal diet to produce experimental diets containing 300, 600 and 900 mg/kg NiCl_2_, respectively.

### 2.2. Detection of the Splenocyte Apoptosis by TUNEL and FCM

#### 2.2.1. TUNEL Assay

Five broilers in each group were euthanized at 14, 28 and 42 days of age for gross examination. Spleens were removed, fixed in 4% neutral buffered paraformaldehyde, and embedded in paraffin. The terminal dexynucleotidyl transferase (TdT)-mediated dUTP nick end labeling (TUNEL) assay was performed in the deparaffinized section (5 μm thick) with an apoptosis detection kit (EMD Biosciences, Inc. La Jolla, CA, USA) according to the manufacturer’s instructions. Briefly, splenic slices were rehydrated in a series of xylene and ethanol solutions and then incubated in a humidified chamber at room temperature for 20 min with proteinase K (Cat. No: JA 1477, EMD Biosciences, Inc. La Jolla, CA, USA). Slices were then rinsed with tris-buffered saline (TBS). The entire specimens were covered with 3% H_2_O_2_ and then incubated at room temperature for 5 min. Slices were rinsed with TBS. TUNEL enzyme (Cat. No. JA 1559, EMD Biosciences, Inc. La Jolla, CA, USA) and label solution (Cat. No. JA 1560, EMD Biosciences, Inc. La Jolla, CA, USA) were mixed and applied to slices, which were incubated again in the humidified chamber for 1 h at 37 °C. Slices were thoroughly rinsed with TBS. Stop buffer, block buffer, and conjugate were applied in turn. Diaminobenzidine solution was applied for 10–15 min to stain the nuclei of apoptotic cells. The methyl green solution was used to counterstain the nuclei of normal cells. Slices were dehydrated in a series of three ethanol baths and twice xylene baths, 5 min for each. The positive cells were quantified using Image-Pro Plus 5.1 (Media Cybernetics, Inc. Bethesda, MD, USA). Five slices were measured in each group and five microscopic areas (randomly chosen) were measured in each slice. Then the numbers were averaged. 

#### 2.2.2. Annexin V Apoptotic Detection by FCM

At 14, 28 and 42 days of age, five broilers in each group were humanely killed, and then the spleens were removed immediately and ground to form a cell suspension, which was then filtered with 300-mesh nylon screen. The cells at a concentration of 1×10^6^ cells/mL were washed twice with cold PBS (phosphate buffer solution, pH 7.2-7.4), and suspended in 1×binding buffer (Cat. No: 51-66121E, BD, Co. Ltd. San Diego, CA, USA). 100 μL of the cell suspension was transferred to a 5ml culture tubes, and then 5 μL of Annexin V-FITC (Cat. No: 51-65874X, BD, Co. Ltd. San Diego, CA, USA) and 5 μL of PI (Cat. No: 51-66211E) were added. The mixture was gently swirled to misce bene and incubated for 15 min at 25 °C in the dark. 400 μL of 1× binding buffer was added to each tube, and finally analysis by FCM (BD FACS Calibur, BD, Co. Ltd. San Diego, CA, USA) was conducted within 1 h. 

### 2.3. Determination of Bax, Bcl-2 and Caspase-3 in the Spleen

#### 2.3.1. Detection of Bax, Bcl-2 and Caspase-3 mRNA Expression Levels by qRT-PCR

At the age of 14, 28 and 42 days, spleens of five broilers in each group were removed and immediately stored in liquid nitrogen. Adding liquid nitrogen into a mortar, the spleens were ground into a homogenized powder with a pestle. The powder was filled into EP tubes immediately, then, stored at −80 °C for future usage.

Total RNA was extracted from the powder of the spleen by RNAiso Plus (9108/9109, Takara Tomy, Tokyo, Japan). The mRNA was then reverse transcribed into cDNA using PrimScript^TM^ RT reagent kit with gDNA Eraser (RR047A, Takara). The cDNA was used as a template for qRT-PCR analysis. Sequence of primers was obtained from GenBank of NCBI. Primers were designed with Primer 5, and synthesized by BGI Tech (Shenzhen, China) (Table 1).

For qRT-PCR reactions, 25 μL mixture was made by using SYBR^®^ Premix Ex Taq^TM^ Ⅱ (DRR820A, Takara), containing 12.5 μL Tli RNaseH Plus, 1.0 μL of forward and 1.0 μL of reverse primer, 8.5 μL RNAase-free waters and 2 μL cDNA. Reaction conditions were set to 3 min at 95 °C for 1 cycle, followed by 44 cycles of, 30s at Tm of a specific primer pair, followed by 1 cycle of 10 s at 95 °C, 72 °C for 10 s, and 10 s at 95°C, using Thermal Cycler (C1000, Bio-Rad, Hercules, CA, US). β-actin was used as an internal control gene. Results were analyzed with the method of 2 ^-ΔΔCT^.

**Table 1:** Nucleotides used as primers in qRT-PCR analysis of mRNA expression in the spleen.

Primer		Sequence (5’→3’)	Accession Number
Bax	F	TCCTCATCGCCATGCTCAT	XM_422067
	R	CCTTGGTCTGGAAGCAGAAGA	XM_422067
Bcl-2	F	GATGACCGAGTACCTGAACC	NM_205339
	R	CAGGAGAAATCGAACAAAGGC	NM_205339
Caspase-3	F	TGGCCCTCTTGAACTGAAAG	NM_204725
	R	TCCACTGTCTGCTTCAATACC	NM_204725
β-actin	F	TGCTGTGTTCCCATCTATCG	L08165
	R	TTGGTGACAATACCGTGTTCA	L08165

#### 2.3.2. Detection of Bax, Bcl-2 and Caspase-3 Contents by ELISA

At 14, 28 and 42 days of age, spleens of five broilers from each group were humanely removed. Then spleens were immediately removed, and chilled to 0 °C in 0.85% NaCl solution. The spleens were rinsed with icy isotonic saline (0.9% wt/vol NaCl) before being weighed. A 10% homogenate of spleen was produced by using glass homogenizers and centrifugation at 3,000 × g for 10 min at 4 °C. The supernatant was conserved for further analysis. The concentrations of Bax, Bcl-2, Caspase-3 were assayed by ELISA Kit for chicks (MyBioSource, Inc. San Diego, CA USA) [51]. Bax (REF: MBS043584), Bcl-2 (REF: MBS260943) and Caspase-3 (REF: MBS023920). The contents of Bax, Bcl-2 and Caspase-3 were determined by the standard curve and were expressed as nanograms per milliliter. 

### 2.4. Detection of Oxidative Stress Parameters in the Spleen

Preparation of the splenic homogenate was described in Section 2.3.2 above. After determining the amount of total protein in the supernatant of splenic homogenate with the Bradford method (a rapid and accurate method for the estimation of protein concentration, which relies on the binding of the dye Coomassie Blue G250 to protein) [52], the SOD, CAT and GSH-Px activities, and ability to inhibit hydroxyl radical, and MDA and GSH contents in the supernatant were detected according to the instruction of the reagent kits (SOD: Cat. No: A001-1, LOT: 201211; CAT: Cat. No: A007, LOT: 201211; GSH-Px: Cat. No: A005, LOT: 201211; abilities to inhibit hydroxyl radical: Cat. No: A018, LOT: 201211; GSH: Cat. No: A006, LOT: 201211; MDA: Cat. No: A003-2, LOT: 201211; total protein: Cat. No: A045-2, LOT: 201211, Nanjing Institute of Jiancheng Biological Engineering, Nanjing, China). The absorbance of SOD, CAT, GSH-Px, abilities to inhibit hydroxyl radical, MDA, GSH and total protein were measured at 550, 240, 412, 550, 532, 420 nm and 590 nm respectively, and data were gained by using a microtiter plate reader (Thermo, Waltham, MA, USA). 

### 2.5. Statistical Analysis

The significance of difference among four groups was analyzed by variance analysis. The analysis was performed using one-way analysis of variance (ANOVA) test of SPSS 16.0 for Windows, and results were presented as means ± standard deviation (*X* ± SD). A value less than 0.05 (*p* < 0.05) was considered significant when compared to the control group.

## 3. Results

### 3.1. Changes of Splenocyte Apoptosis

**Figure 1:**
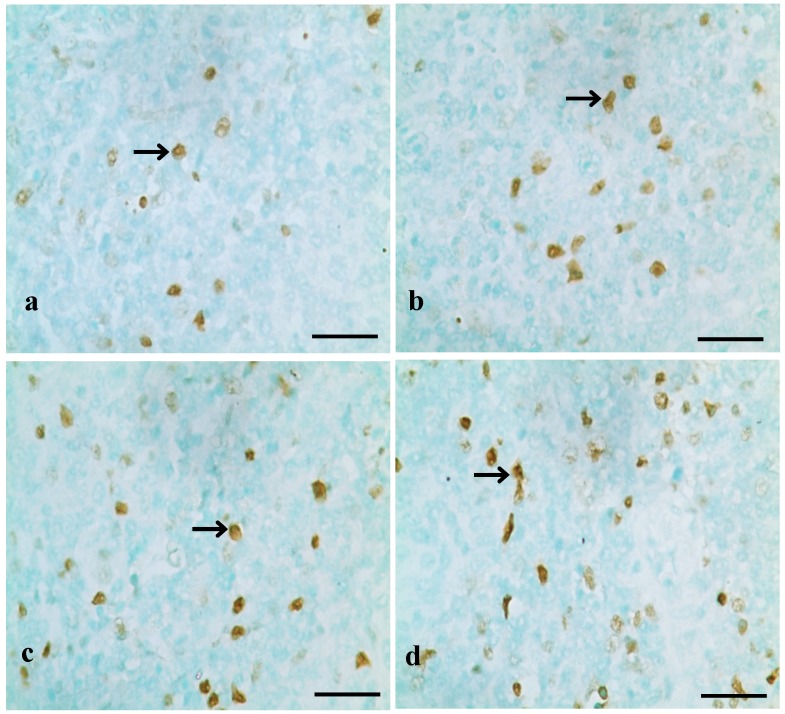
Positive cells stained by TUNEL in the spleen at 14 days of age.

Notes: a. Control group. There are a few positive cells (brown-stained, TUNEL, 1,000 ×, bar = 20 ìm); b. The 300 mg/kg group. The numbers of positive cells are slightly increased; c–d. 600 and 900 mg/kg groups. The numbers of positive cells are significantly increased.

**Figure 2:**
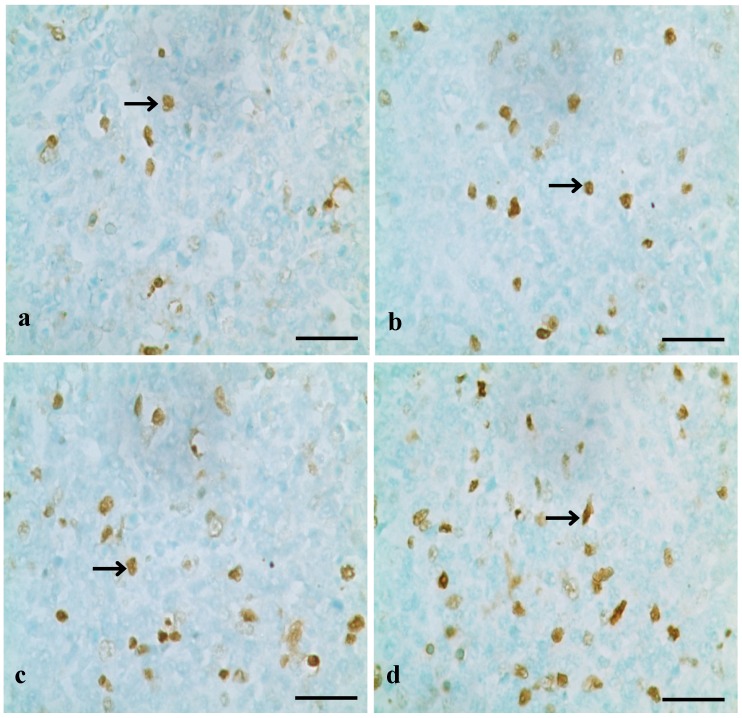
Positive cells stained by TUNEL in the spleen at 28 days of age.

Notes: a. Control group. There are a few positive cells (brown-stained) (TUNEL, 1000×, bar = 20 ìm). b. The 300 mg/kg group. The numbers of positive cells are increased; c–d. The 600 and 900 mg/kg groups. The numbers of positive cells are significantly increased.

**Figure 3:**
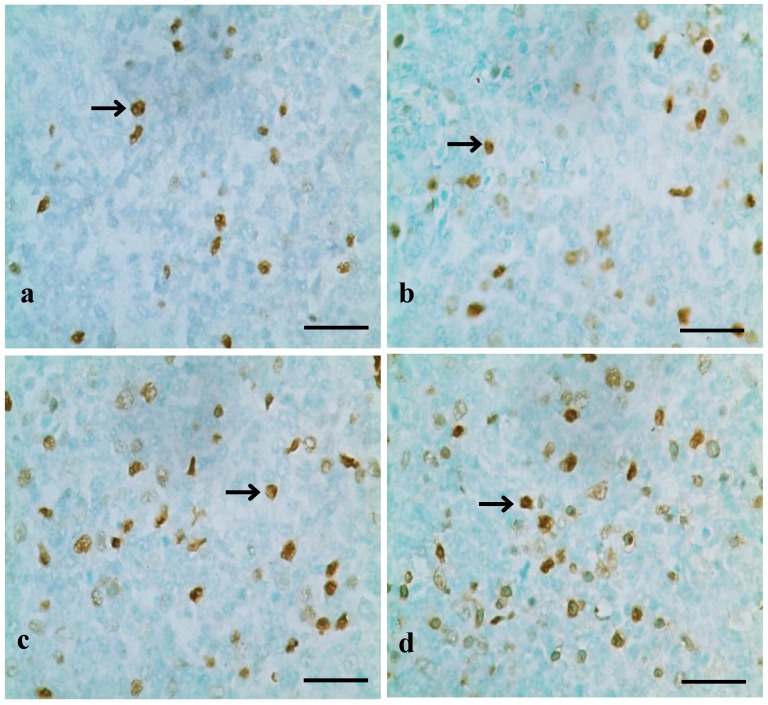
Positive cells stained by TUNEL in the spleen at 42 days of age.

Notes: a. Control group. There are a few positive cells (brown-stained) (TUNEL, 1000×, bar = 20 ìm); b–d. The 300, 600 and 900 mg/kg groups. The numbers of positive cells are significantly increased.

**Figure 4:**
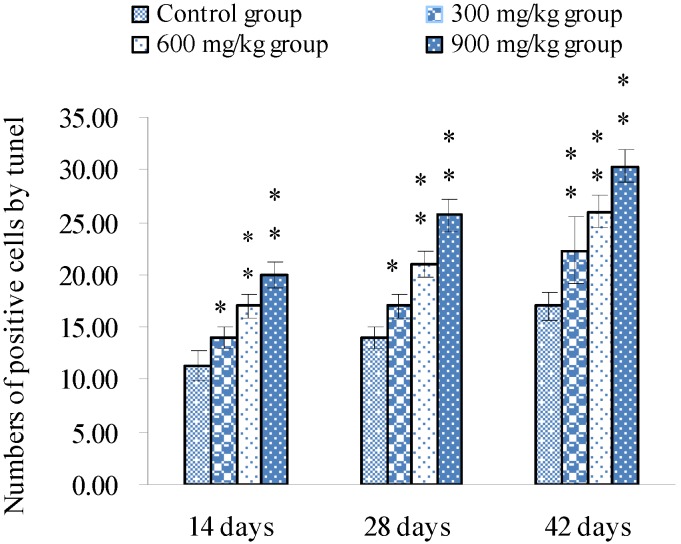
Numbers of positive cells by TUNEL.

TUNEL assays showed that the nuclei of positive cells were stained in brown (Figure 1, Figure 2 and Figure 3). The results in Figure 4 show that the number of apoptotic splenocytes was significantly higher (*p* < 0.05 or *p* < 0.01) in the 300 mg/kg, 600 mg/kg and 900 mg/kg groups from 14 to 42 days of age than those in the control group. 

**Figure 5:**
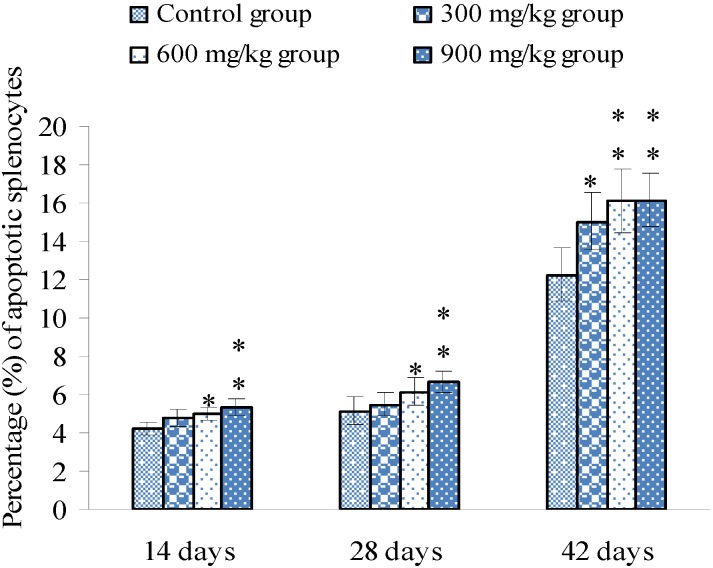
Percentage (%) of apoptotic splenocytes by FCM.

Notes: Data are presented with the means ± standard deviation (n = 5 × 5), * *p* < 0.05, ** *p* < 0.01, compared with the control group. Data were analyzed by the variance (ANOVA) test of the SPSS 16.0 software.

Apoptosis detection by FCM showed that the percentages of apoptotic splenocytes were significantly increased (*p* < 0.05 or *p* < 0.01) in the 600 and 900 mg/kg groups from 14 to 42 days of age, and in the 300 mg/kg group at 42 days of age when compared with those of the control group, as shown in Figure 5. 

### 3.2. Changes of Bax, Bcl-2 and Caspase-3 mRNA Expression Levels and Contents in the Spleen

**Figure 6:**
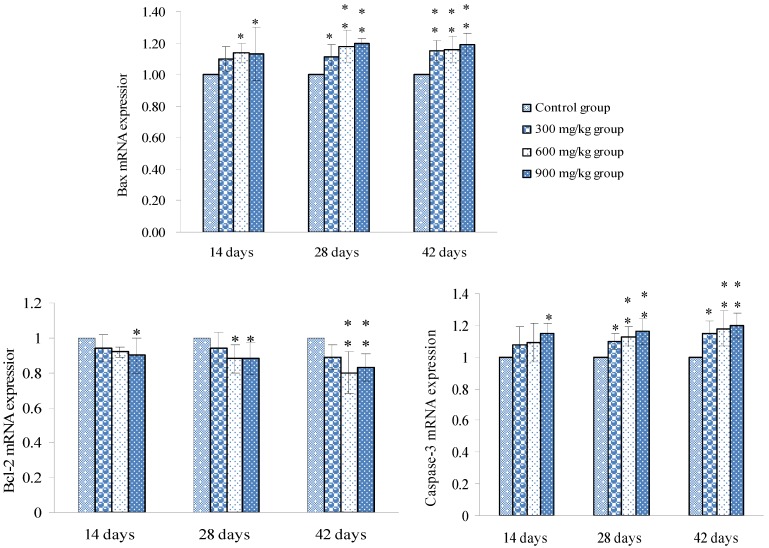
mRNA expression levels of Bax, Bcl-2 and Caspase-3 in the spleen.

Notes: Data are presented with the means ± standard deviation (n = 5 × 5), * *p* < 0.05, ** *p* < 0.01, compared with the control group. Data were analyzed by the variance (ANOVA) test of the SPSS 16.0 software.

**Figure 7:**
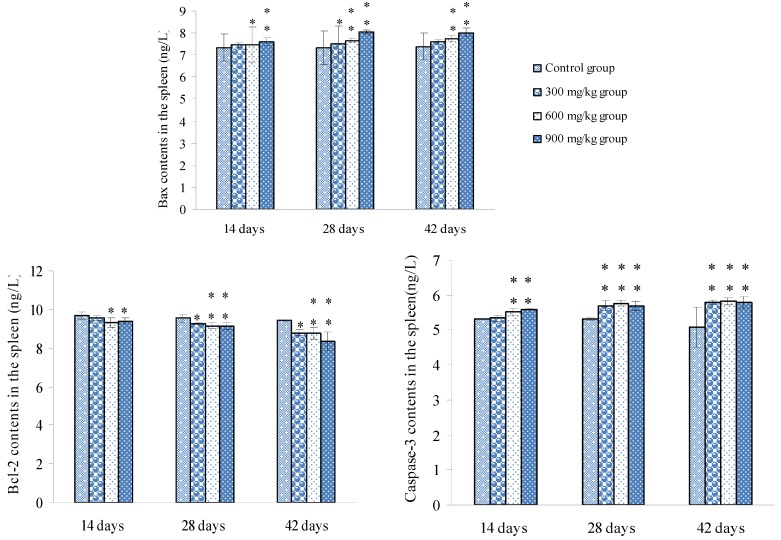
Changes of Bax, Bcl-2 and Caspase-3 contents in the spleen.

Notes: Data are presented with the means ± standard deviation (n = 5 × 5), * *p* < 0.05, ** *p* < 0.01, compared with the control group. Data were analyzed by the variance (ANOVA) test of the SPSS 16.0 software.

Changes of Bax, Bcl-2 and Caspase-3 mRNA expression levels were shown in Figure 6, and changes of Bax, Bcl-2 and Caspase-3 contents were shown in Figure 7. 

### 3.3. Changes of the Oxidative Stress Parameters in the Spleen

**Figure 8:**
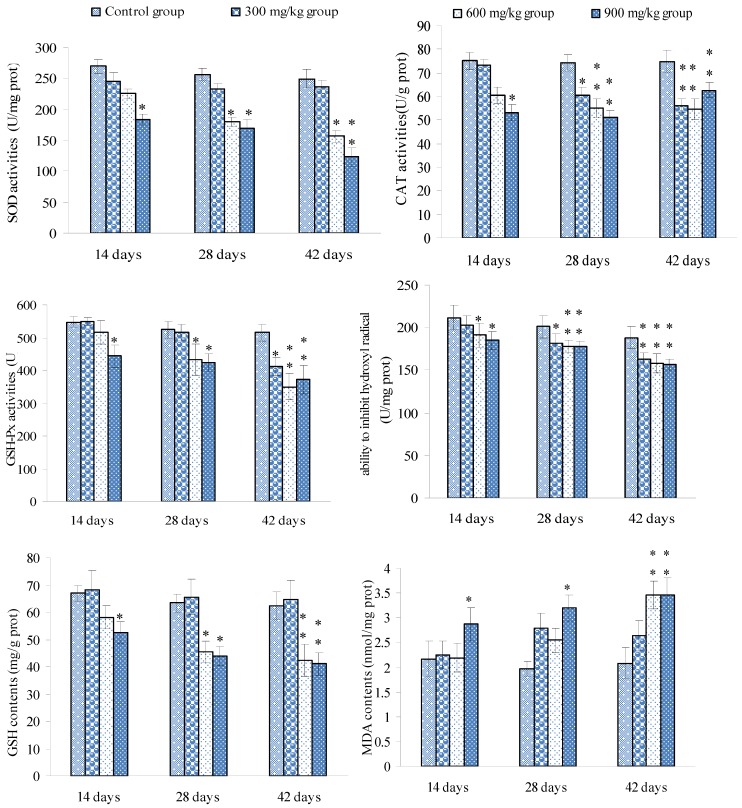
Changes of the oxidative stress parameters in the spleen.

Notes: Data are presented with the means ± standard deviation (n = 5 ×5), * *p* < 0.05, ** *p* < 0.01, compared with the control group. Data were analyzed by the variance (ANOVA) test of the SPSS16.0 software.

Changes of the SOD, CAT and GSH-Px activities, the ability to inhibit hydroxyl radical, GSH and MDA contents were shown in Figure 8. 

## 4. Discussion

At present, very limited studies focus on NiCl_2_-induced apoptosis and oxidative stress in the spleen of animals and human. Thus, the aim of this study is to investigate the oxidative stress, alternations of Bax, Bcl-2 and Caspase-3 mRNA expression levels and contents, and apoptosis in the spleen of broilers induced by dietary NiCl_2_, and to reveal the association between splenocyte apoptosis and alternations of Bax, Bcl-2 and Caspase-3 mRNA expression levels and contents, and oxidative stress, and to provide new experimental evidence to clarify the mechanism of NiCl_2_ toxicity on spleen and splenic functions.

Apoptosis is the programmed cell death in eukaryotes, which is essential for development and tissue homeostasis by providing a protective mechanism to clean out aged or damaged cells [53,54]. It can be activated by various stimuli, including ultraviolet (UV) irradiation and serum starvation [55]. Cell death through apoptosis is tightly controlled by changes, interactions and post-translational modifications (including proteolytic cleavage and phosphorylation) of proteins [56]. In the present study, the results of FCM and TUNEL assays showed that dietary NiCl_2_ in excess of 300 mg/kg could increase the percentage of splenocyte apoptosis, which was consistent with the alternations of Bax, Bcl-2 and Caspase-3 mRNA expression levels and contents in the spleen due to the close relationship between apoptosis and apoptosis proteins.

Apoptosis mainly has two pathways: the intrinsic and the extrinsic ones. The former involves initial mitochondrial perturbation resulting from cellular stress or cytotoxicity [55,57,58]. The Bcl-2 protein family is the major regulators and effectors of the intrinsic pathway [59]. Bcl-2 locates mainly on the outer membrane of mitochondria, and its overexpression protects cells from apoptosis caused by various stimuli. The Bcl-2 family can be categorized into the anti-apoptotic proteins (Bcl-2-like proteins such as Bcl-2 and Bcl-X_L_), and the pro-apoptotic proteins (such as Bax-like and the BH3-only proteins) [59]. The activation of the pro-apoptotic proteins will induce cytochrome c release from mitochondria into the cytoplasm [60,61,62,63]. However, Bcl-2-like proteins can prevent Bax-induced cell death by blocking cytochrome c release [53]. The increase in Bax mRNA expression levels and contents and the decrease in Bcl-2 mRNA expression levels and contents in our study caused the cytochrome c release and apoptosis initiation in the spleen. Caspase is a family of single-chain synthesized zymogens playing central roles in apoptotic signaling and execution [64,65]. The executor-Caspase-3 can be activated both by the intrinsic and the extrinsic apoptotic pathways. In the intrinsic one, cytochrome c enters the cytoplasm, and activates a chain-reaction of caspase family and the cell demise [66]. It is reported that Ni (II)-induced ROS can activate Caspase-3 expression, increase Caspase-3-like protease activity, and then cause cell death in mice [34,35]. The results showed that the significantly increased Caspase-3 mRNA expression levels and contents in the NiCl_2_-added groups promoted apoptosis in the spleen.

There are enzymatic and non-enzymatic internal defense systems to prevent the oxidative stress in the body [67]. Endogenous antioxidants such as SOD, GSH-Px, CAT and GSH, and the ability to inhibit hydroxyl radical are important bio-markers of the anti-oxidative system. These endogenous antioxidants are able to inhibit overproduced free radicals (including ROS), and avoid excess lipid peroxidation. SOD can break up ROS and repair cellular damage caused by ROS. Ni ion accumulation may be responsible for the generation of ROS and the enhancement of LPO [23], and NiCl_2_ is related to DNA oxidation and DNA strand breaks in rat’s liver [23]. Hydroxyl radical can affect cellular structure protectors (such as CAT, SOD and GSH) [68]. Additionally, Ni can enhance lipid peroxidation (LPO) and caused cellular damage and reduced GSH contents, and CAT and GSH-Px activities in the liver and kidney of rats [24,25]. GSH is considered to be an important bio-marker in LPO and is important in maintaining the cellular redox status [67]. Reduction of GSH is a marker of oxidative stress [21,69] and can significantly enhance NiCl_2_-induced apoptosis [70]. Thus, the reduced activities of GSH-Px and CAT may be closely associated to the reduction of GSH contents. The interaction of the antioxidants may be the main reason which caused the reduction of activities of SOD, CAT and GSH-Px and the decreased content of GSH as shown in Figure 8. 

Free radicals (including ROS and ∙OH) are products of metabolic reactions and byproducts of immune reactions, and can damage cellular lipids, proteins and DNA [71]. LPO may be a contributing factor in Ni-induced tissue oxidative stress [22]. In the LPO process, free radicals attack lipids, and abstract electrons from the cell membrane through a chain reaction [63,72], and cause the generation of MDA (Figure 8), which inhibits activity of antioxidants. LPO can reduce membrane fluidity and integrity, and increase membrane fragility [73,74]. There is no doubt that NiCl_2_-induced oxidative stress to the spleen impairs the splenic function. 

The changes of the abovementioned oxidative stress parameters showed that NiCl_2_ caused oxidative damage in the spleen, which is consistent with the increased percentages of splenocyte apoptosis. Previous studies have revealed that ROS and oxidative damage are strongly related to the induction of apoptosis [67]. Thus, the results in the present study revealed the NiCl_2_-induced oxidative damage in the spleen caused alterations of Bax, Bcl-2 and Caspase-3 mRNA expression levels and contents, and finally resulted in splenocyte apoptosis *via* the intrinsic apoptosis pathway as well as oxidative damage. 

## 5. Conclusions

In conclusion, dietary NiCl_2_ in excess of 300 mg/kg causes apoptosis, alters Bax, Bcl-2 and Caspase-3 mRNA expression levels and contents, and results in oxidative stress in the spleen of broilers. Also, splenocyte apoptosis is closely related, to not only the alternations of Bax, Bcl-2 and Caspase-3 mRNA expression levels, but also oxidative stress. Consequently the splenic immunity and blood filtration functions are impaired in broilers. This study provides new experimental evidences and an animal model for further understanding the mechanism of the effects of NiCl_2_ on the spleen.
